# The interaction between depression diagnosis and BMI is related to altered activation pattern in the right inferior frontal gyrus and anterior cingulate cortex during food anticipation

**DOI:** 10.1002/brb3.2695

**Published:** 2022-08-12

**Authors:** A Manelis, YO Halchenko, S Satz, R Ragozzino, S Iyengar, HA Swartz, MD Levine

**Affiliations:** ^1^ Department of Psychiatry University of Pittsburgh Pittsburgh Pennsylvania; ^2^ Department of Psychological and Brain Sciences Dartmouth College Hanover New Hampshire; ^3^ Department of Statistics University of Pittsburgh Pittsburgh Pennsylvania

**Keywords:** anterior cingulate cortex, anticipation, BMI, depression, fMRI, food, overweight/obese, right inferior frontal gyrus

## Abstract

**Background:**

Depression and overweight/obesity often cooccur but the underlying neural mechanisms for this bidirectional link are not well understood.

**Methods:**

In this functional magnetic resonance imaging study, we scanned 54 individuals diagnosed with depressive disorders (DD) and 48 healthy controls (HC) to examine how diagnostic status moderates the relationship between body mass index (BMI) and brain activation during anticipation and pleasantness rating of food versus nonfood stimuli.

**Results:**

We found a significant BMI‐by‐diagnosis interaction effect on activation in the right inferior frontal gyrus (RIFG) and anterior cingulate cortex (ACC) during food versus nonfood anticipation (*p* < .0125). Brain activation in these regions was greater in HC with higher BMI than in HC with lower BMI. Individuals with DD showed an opposite pattern of activation. Structural equation modeling revealed that the relationship between BMI, activation in the RIFG and ACC, and participants’ desire to eat food items shown in the experiment depended on the diagnostic status.

**Conclusions:**

Considering that food anticipation is an important component of appetitive behavior and that the RIFG and ACC are involved in emotion regulation, response inhibition and conflict monitoring necessary to control this behavior, we propose that future clinical trials targeting weight loss in DD should investigate whether adequate mental preparation positively affects subsequent food consumption behaviors in these individuals.

## 1 INTRODUCTION

Depressive disorders (DD) (Judd et al., 2000) and overweight/obesity (Afshin et al., 2017) are important personal and public health problems. Over 264 million people worldwide suffer from depression (https://www.who.int/news‐room/fact‐sheets/detail/depression), and over 1.9 billion adults are overweight (https://www.who.int/news‐room/fact‐sheets/detail/obesity‐and‐overweight). DD and overweight/obesity often cooccur (Jantaratnotai et al., 2017) and may share common biological pathways (Milaneschi et al., 2019). Being overweight/obese increases the risk of developing mood and anxiety disorders (Antoniou et al., 2017; Luppino et al., 2010; Mannan et al., 2016) and predicts more severe mood symptoms (Opel et al., 2015), while weight loss is associated with improvements in depression symptoms (Burgmer et al., 2014; Mitchell et al., 2014). In the opposite direction, current mood state is found to influence motivation for food reflected by increased subjective appetite and attentional bias toward food cues (Hepworth et al., 2010). The bidirectional link between depression and obesity may be explained by factors including inflammatory and metabolic mechanisms (Patist et al., 2018) as well as functioning of systems involved in homeostatic adjustments (Pariante & Lightman, 2008) and integration of homeostatic responses and emotion regulation (Milaneschi et al., 2019). Understanding the neurobiological factors that contribute to the bidirectional relationship between DD and unhealthy weight is critical for development of prevention and intervention treatments.

It was shown that cognitive function is reduced in individuals with DD relative to HC (Clark et al., 2009), more so in individuals with DD and obesity than in those with DD and normal weight (Hidese et al., 2018). This effect may be related to aberrant brain structure and function in individuals with overweight/obesity and those with DD. For example, lower grey matter volume was found in both individuals with higher BMIs (García‐García et al., 2019; Hamer & Batty, 2019) and individuals with DD (Wise et al., 2016). Noteworthy, grey matter volume in the prefrontal and temporal cortices was lower in individuals with DD who had higher BMI (Hidese et al., 2018; Opel et al., 2015) and in those with more severe course of DD (Opel et al., 2015). The findings regarding the differences between obese and lean individuals for food versus nonfood pictures are inconsistent. One meta‐analysis did not identify significant differences between these two groups (Morys et al., 2020); however, the other meta‐analysis determined that individuals with obesity, compared to those with normal weight, had greater activation in the left dorsomedial prefrontal, right inferior frontal (RIFG), superior frontal, anterior cingulate cortices (ACC), and parahippocampal gyri, but lower activation in the left dorsolateral prefrontal and insular cortices for food versus nonfood stimuli (Brooks et al., 2013). Greater activation in the insula, striatum, and fusiform gyrus predicted less successful longitudinal outcome in the weight maintenance program (Murdaugh et al., 2012). Many of these same regions (e.g., the ACC, insula, amygdala, prefrontal cortices) showed aberrant activation patterns in individuals with DD versus HC (Hamilton et al., 2012; Li & Wang, 2020) with the insula showing distinct activation patterns as a function of appetite decrease/increase in individuals with DD in response to the pictures of food (Simmons et al., 2016; Simmons et al., 2020). The precuneus and ACC showed stronger activation in obese individuals with DD and obese HC during word pleasantness judgments (Restivo et al., 2020).

Appetitive behavior starts before food encounters and consumption and includes a food anticipation phase when no sensory information about food (visual image, smell, etc.) is yet available. For example, one might think “I wish I could eat a steak right now” in the middle of a meeting despite the absence of direct food cues. Cognitive and neural processes associated with food anticipation can motivate participants to obtain and consume food even when they are not hungry (Colagiuri & Lovibond, 2015). While motivational processes elicited during food anticipation may benefit cognitive functioning—anticipating food prior to performing on a cognitive test improved test performance in overweight/obese but not lean individuals (Segovia et al., 2019)—they can also lead to food overconsumption (Colagiuri & Lovibond, 2015) and eventual weight gain. Anticipation of food‐related reward was linked to greater activation in the ACC and parahippocampal gyrus in emotional eaters (Bohon et al., 2009) and in the ventral striatum in hungry individuals (Simon et al., 2017). Lower ventral striatal activation during anticipation of food‐related reward was associated with greater weight loss during dieting (Simon et al., 2018). In addition to hunger and weight status, mood disorders also may affect anticipatory processing. For example, activation patterns in the ACC, prefrontal, parietal, and ventral striatal regions distinguished individuals with mood disorders from HC during anticipation of emotional stimuli (Manelis et al., 2016; Manelis et al., 2020). Taken together, these findings suggest that both participants’ diagnostic status (DD vs. HC) and their BMI may contribute to neurobiology underlying food anticipation. Although brain responses during food anticipation could be an important predictor of subsequent appetitive behavior, it remains unclear how depression and BMI are linked to anticipatory brain activation.

We examined the interaction effect of diagnostic status and BMI on anticipatory brain activation in a task involving anticipation of food and nonfood items and then, judging the pictures of food and nonfood items as pleasant or unpleasant. Inclusion of nonfood items was necessary to establish an activation baseline for visual and cognitive processing. While the World Health Organization provides standard BMI categories, the threshold to label individuals as overweight/obese is subjective, “ill‐defined” (Nuttall, 2015), and may change over time to reflect current BMI distributions in the population, mortality rates associated with obesity, and other factors (Ahima & Lazar, 2013; Hothorn et al., 2017; Nuttall, 2015). To avoid potential biases of BMI categorization, we followed the Research Domain Criteria approach (Insel et al., 2010) and examined BMI as a continuous variable. We hypothesized that, during anticipation of food versus nonfood pictures, diagnostic status would moderate the relationship between BMI and brain activation in the regions sensitive to food versus nonfood stimuli in normal weight versus obese individuals (Brooks et al., 2013). Considering impaired emotion regulation in individuals with DD (Joormann & Gotlib, 2010), anticipating food stimuli might be more distressing for individuals with DD and high BMI than to other participants due to their reduced ability to inhibit food consumption. This may result in reduced activation in prefrontal cortical brain regions responsible for emotion regulation. The relationship between anticipatory brain responses associated with food picture processing, BMI and ratings of how much participants liked and wanted to eat food items presented in the study was examined using structural equation modeling. These models provided additional insight into brain‐behavior response to food versus nonfood stimuli in individuals with DD and HC along BMI measures.

## 2 METHOD AND MATERIALS

### 2.1 Participants

The study was approved by the University of Pittsburgh Institutional Review Board. Participants were recruited from the community, local universities, and medical centers. Written informed consent was obtained from all participants. HC had no personal or family history of psychiatric disorders. Symptomatic individuals met DSM‐5 criteria for major depressive or persistent depressive disorders that are referred here collectively as depressive disorders (DD). Individuals with depressive disorders were recruited across mood states. All participants were right‐handed and fluent in English. The groups were matched on age, sex, and BMI. We scanned 113 participants (53 HC and 60 DD). Of them, 5 HC and 6 DD were removed from the analyses due to excessive motion (>4 mm between the fMRI volumes in any direction), scanning artifacts, or more than 20% of missing responses on the task, thus leaving 102 participants (48 HC and 54 DD) in the analyses. Participants were schedule to arrive to the University of Pittsburgh/UPMC Magnetic Resonance Research Center 60–90 min before the scan to complete practice trials, change to the MRI compatible clothes, and complete the set up for the scan. During this time, participants were not allowed to eat or drink anything but water.

### 2.2 Clinical assessment

All diagnoses were made by a trained clinician and confirmed by a psychiatrist according to DSM‐5 criteria using SCID‐5 (First et al., 2015). We also assessed current depression symptoms (Hamilton Rating Scale for Depression; HRSD‐25; Hamilton, 1960), current mania symptoms (Young Mania Rating Scale; YMRS; Young et al., 1978), and lifetime dimensional symptoms of depression (Moods Spectrum self‐report questionnaire; MOODS‐SR; Dell'Osso et al., 2002). A total psychotropic medication load was calculated for each participant (Hassel et al., 2008; Manelis et al., 2016). Exclusion criteria included a history of head injury, metal in the body, pregnancy, claustrophobia, neurodevelopmental disorders, systemic medical illness including any metabolic or endocrine disorders (e.g., diabetes, hypo/hyperthyroidism, hypertension), premorbid IQ < 85 per the National Adult Reading Test (NART; Nelson, 1982), current alcohol/drug abuse, YMRS scores > 10 at scan, meeting criteria for any psychotic‐spectrum disorder. Participants were excluded from the study if they were on steroid, opioid, or thyroid medications as well as the medications for high blood pressure or cholesterol.

### 2.3 Behavioral assessments

Participants rated their feelings toward food (i.e., hunger, fullness, urge to eat) prescan using a 6‐question Visual Analogues Scale (VAS) ranging from 0 (“Not at all”) to 100 (“Extremely”). These data were only available for 33 HC and 36 DD.

**FIGURE 1:**
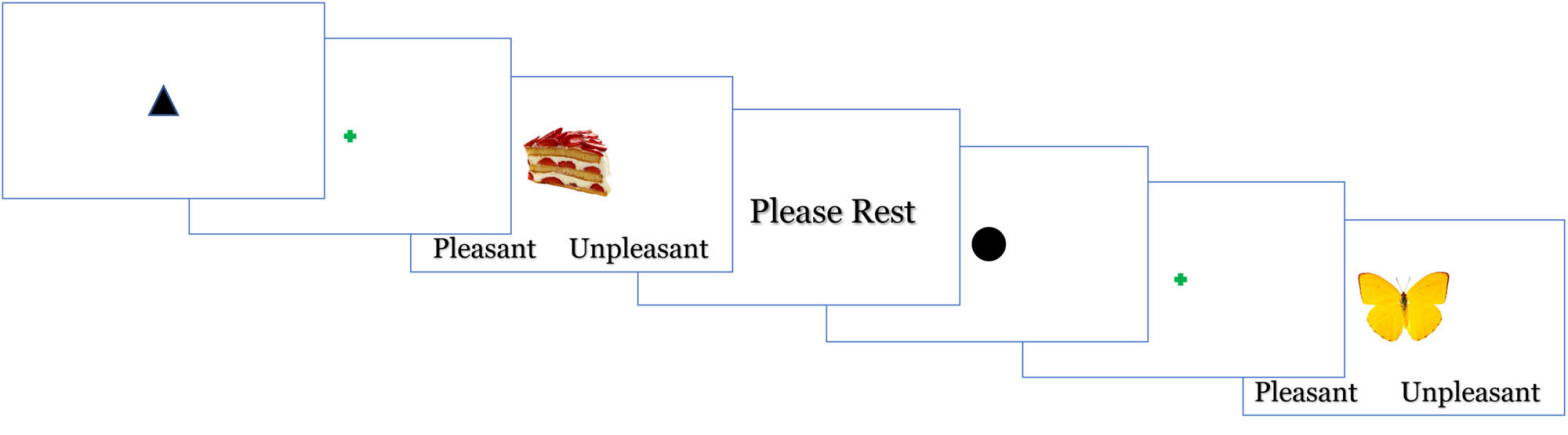
An example of the food and object trials in the Food and Object Cued Encoding Task

Inside the scanner, participants performed a Food and Object Cued Encoding Task (Figure 1). Each trial of this task started with a 4‐s *anticipation* phase during which participants were presented with either a triangle predicting food or a circle predicting object categories of pictures. During practice trials, participants learned the association between abstract shapes and stimulus categories as well as the instructions to mentally prepare for the category of items predicted by the cue. After the cue, a stimulus from the predicted category was presented for the length of the response time but no longer than 1.5 s. All stimuli were taken from the *Food‐pics* image database (Blechert et al., 2014). Participants rated the stimulus as pleasant or unpleasant by pressing a corresponding button with the index finger on one hand for pleasant images and on the other hand for unpleasant images. The hand assignment for pleasantness rating was counterbalanced across subjects. A total of 48 trials (50% of food stimuli) were presented over two 4‐min runs. The rest periods between the trials ranged from 3.3 to 4.9 s.

After the scan, participants completed a questionnaire regarding the strategies that they used during mental preparation. Based on a pilot study that used an open‐end questions about anticipation strategies, we generated a list of six strategies: verbal, visual, rule‐related, sensory, memory, and suppression. Specifically, a verbal strategy involved verbalizing the cued category (e.g., repeated the words “food” or “object” when presented with the cues), a visual strategy involved visualizing specific items from the category (e.g., pictured a burger or a baseball bat), a rule‐based strategy involved trying to remember or repeat task‐related rules, a sensory strategy involved imagining a related experience (e.g., imagined the taste of a burger or swinging a baseball bat), a memory strategy involved thinking of a related memory (e.g., getting a burger with friends last week or hitting a home run in 7th grade), and a suppression strategy involved trying to ignore or suppress cued categories of items. Participants could select as many strategies from the list as they wished. If they did not use any of these strategies, participants were given options to indicate that they did not try to prepare themselves for the cued categories of items, or that they did not use any strategies from the list. In the latter case, participants were asked to describe their own strategy in the space provided for each category of items.

After completing the questionnaire regarding anticipation strategies, participants were shown food pictures they had seen during the scan and were asked to indicate on a 9‐point scale (1—do not like/want it, 9—like/want it very much) how much they normally like to eat those items (“LIKE” condition) and how much they wanted to eat them “right now” at the time of the assessment (“WANT” condition).

### 2.4 Neuroimaging data acquisition

The neuroimaging data were collected at the University of Pittsburgh/UPMC Magnetic Resonance Research Center using a 3T Siemens Prisma scanner with a 64‐channel head coil and named according to the ReproIn convention (Visconti di Oleggio Castello et al., 2020). The EPI data were collected in the anterior‐to‐posterior direction using a multiband sequence (factor = 8, TR = 800 ms, resolution = 2 × 2 × 2 mm, FOV = 210, TE = 30 ms, flip angle = 52°, 72 slices, 315 volumes). High‐resolution T1w images were collected using the MPRAGE sequence (TR = 2400 ms, resolution = 0.8 × 0.8 × 0.8 mm, 208 slices, FOV = 256, TE = 2.22 ms, flip angle = 8°). Field maps were collected in the AP and PA directions using the spin echo sequence (TR = 8000, resolution = 2 × 2 × 2 mm, FOV = 210, TE = 66 ms, flip angle = 90°, 72 slices).

### 2.5 Data analyses

#### 2.5.1 Clinical data analysis

Demographic and clinical characteristics were compared between groups using *t*‐ and chi‐square tests. A total psychotropic medication load was calculated for each participant (Hassel et al., 2008; Manelis et al., 2016).

#### 2.5.2 Behavioral data analysis

The available VAS data were compared between groups using a *t*‐test. A mean percent of “pleasant” responses, the differences in the percent of food versus object pictures judged as pleasant, and reaction time (RT) in the encoding task as well as the “LIKE” and “WANT” ratings of food were calculated for each subject. All repeated measures analyses were conducted using a mixed effects model (“lme4” [Bates et al., 2015], “lmerTest” [Kuznetsova et al., 2017], and “psycho” [Makowski, 2018] packages in R). Chi‐square tests were used to determine if the choice of anticipatory strategies depended on participants diagnostic status (DD vs. HC) and BMI category (normal weight, overweight, obese).

#### 2.5.3 Neuroimaging data analysis

##### Preprocessing

The DICOM images were converted to a BIDS (Gorgolewski et al., 2016) dataset using *heudiconv version 0.5.4* (Halchenko et al., 2019). Data quality was examined using *mriqc* 0.15.1 (Esteban et al., 2017). The data were preprocessed using *fmriprep* 20.1.1 (Esteban et al., 2019). Specifically, T1w images were skull‐stripped, brain surfaces were reconstructed using recon‐all (FreeSurfer 6.0.1; Dale et al., 1999), and brain masks were generated. Preprocessing steps included generating a reference volume and its skull‐stripped version using a custom methodology of *fmriprep*, estimating head‐motion parameters with respect to the BOLD reference before any spatiotemporal filtering using mcflirt (FSL 5.0.9; Jenkinson et al., 2002; RRID:SCR_002823), and applying slice‐time correction using *3dTshift* (Cox & Hyde, 1997; AFNI 20160207; RRID:SCR_005927). Fieldmaps were estimated with *3dQwarp* (Cox & Hyde, 1997; AFNI 20160207) based on two spin echo images collected with opposing phase‐encoding directions (i.e., anterior‐to‐posterior and posterior‐to‐anterior). Based on estimated susceptibility distortion, a corrected EPI (echo‐planar imaging) reference was calculated for more accurate coregistration with the anatomical reference. The BOLD reference was coregistered to the T1w reference using *bbregister* (FreeSurfer) (Dale et al., 1999; RRID:SCR_001847) which implements boundary‐based registration (Greve & Fischl, 2009). Coregistration was configured with six degrees of freedom. The BOLD time‐series were resampled onto the *fsaverage* surfaces (FreeSurfer reconstruction nomenclature) and onto their native space by applying a single, composite transform to correct for head‐motion and susceptibility distortions. The BOLD time‐series were resampled into standard space, generating a preprocessed BOLD image in MNI152NLin2009cAsym space. Automatic removal of motion artifacts using independent component analysis (ICA‐AROMA; Pruim et al., 2015) was performed on the preprocessed BOLD after removal of nonsteady state volumes and spatial smoothing with an isotropic, Gaussian kernel of 6 mm FWHM (full‐width half‐maximum). After that, global signals within the CSF and WM were extracted and regressed out from preprocessed BOLD data and high‐pass temporal filter (90‐s cutoff) was applied. All resamplings were performed with a single interpolation step by composing all the pertinent transformations (i.e., head‐motion transform matrices, susceptibility distortion correction when available, and coregistrations to anatomical and output spaces). Gridded (volumetric) resamplings were performed using *antsApplyTransforms* (ANTs), configured with Lanczos interpolation to minimize the smoothing effects of other kernels (Lanczos, 1964).

##### Subject‐level analysis

Subject‐level statistical maps were computed using FSL 6.0.3 installed system‐wide on the workstation with GNU/Linux Debian 10 operating system with NeuroDebian repository (Halchenko & Hanke, 2012). A hemodynamic response was modeled using a gamma function. A subject‐level model included 4 explanatory variables: food cues, object cues, food pictures, and object pictures. To account for individual differences in visual stimuli anticipation, perception, and processing, brain activation during *anticipation* and *pleasantness rating* of objects was used as the baseline for the analyses of *anticipation* and *pleasantness rating* of food. The contrasts of interest included food versus objects during *anticipation* and food versus objects during *pleasantness rating*. Positive differences (increases) show greater activation for food versus objects (food > objects), while negative differences (decreases) show lower activation for food versus objects (food < objects).

##### Group‐level analysis

###### Primary analyses

Based on the previous meta‐analysis (Brooks et al., 2013) that examined the differences in brain responses to food versus nonfood stimuli in normal weight and overweight/obese individuals, we generated a region of interest (ROI) mask composed of the bilateral regions in the superior frontal gyrus, middle frontal gyrus, inferior frontal gyrus pars triangularis, inferior frontal gyrus pars opercularis, precentral gyrus, medial frontal cortex, anterior cingulate and paracingulate gyri, juxtapositional lobule cortex, insular cortex, posterior and anterior portions of parahippocampal gyrus, as well as caudate, putamen, pallidum, and accumbens. To generate the ROI mask, we extracted images of the regions described above from the Harvard‐Oxford Probabilistic cortical and subcortical structural atlases and then combined them into one binarized mask.

The group‐by‐BMI interaction during *anticipation* and *pleasantness rating* of food versus object stimuli was examined using the *swe* (Sandwich Estimator) approach (Guillaume et al., 2014) in the ROI mask described above using nonparametric permutation inference with 5000 permutations, Threshold‐Free Cluster Enhancement (TFCE) correction, and the FWE‐corrected *p* values threshold setup to *p* = .0125 (or 0.05/4) to Bonferroni correct for the two conditions of interest (*anticipation* and *pleasantness rating*) and 2‐tailed test (i.e., activation increase/decrease). Age, gender, and IQ were used as covariates to account for potential individual differences associated with these variables. Functional localization was determined using the Harvard‐Oxford cortical and subcortical structural atlases and visualized using *fslviewer*. The mean percent signal changes were extracted from the clusters of voxels showing a significant interaction effect and used in the follow‐up analyses.

##### Exploratory analyses

###### The effect of clinical variables on brain activation

We explored the effect of medication load, age of illness onset, number of depressive episodes, and appetite during the past week (as indicated in the HDRS‐25) on the primary findings in the DD group. Appetite was ranked on the scale between “−2” and “2” with “−2” showing significant decrease in appetite base on the HDRS‐25, “−1” mild decrease in appetite, “0” no change in appetite, “1” mild increase in appetite, and “2” significant increase in appetite.

###### Graph modeling approach

To characterize the relationship between BMI, food versus object anticipation and pleasantness rating, as well as the “LIKE” and “WANT” ratings of food and the percent of pleasant items in food versus object categories in individuals with DD and HC, we employed a graph modeling approach using the TETRAD software suite version 6.9.0 (https://github.com/cmu‐phil/tetrad; Ramsey et al., 2010). We used an optimized and parallelized version of the Fast Greedy Equivalence Search (FGES; Chickering, 2002; Meek, 1997; Ramsey et al., 2017) to discover a completed partially directed acyclic graph (DAG) that qualitatively characterizes the data. The FGES uses the input data and background knowledge to search a set of causal Bayesian networks to return the model with highest Bayesian score. The following background knowledge was specified to constrain some connectivity directions: BMI is a trait characteristic that cannot be affected by brain activation during the task; therefore, the connections could go from the node denoting BMI to any node denoting brain activation, but not the other way around. A stimulus anticipation always preceded in time a stimulus presentation and pleasantness rating. Therefore, the connections could go from the nodes denoting anticipatory brain activation to the nodes denoting brain activation during pleasantness rating, but not the other way around. While, theoretically, brain activation during pleasantness rating on trial “*n*” could affect anticipatory brain activation on the trial “*n* + 1,” in practice, the order of the trials was randomized and unique for each subject, so the effects described above would reflect noise rather than systematic causal relationships. The rating of how much participants liked (“LIKE” rating) or wanted (“WANT” rating) food items shown during the experiment as well as the differences in the percent of food versus object items judged pleasant could be associated to participants general attitude toward food, brain activation during the task, BMI, and other factors; therefore, we did not restrict the connectivity directions for these variables and allowed the FGES algorithm to determine them. After the FGES discovered the completed partially DAGs for each diagnostic group, DAGs were selected in the equivalence class, and structural equation modeling (SEM) parametric model was applied to quantitatively characterize the DAG models. The SEM parametric model was estimated using the regression optimizer and the full Information Maximum Likelihood (FIML) score. The model coefficients, as well as the standard errors, *t*‐statistics, and *p* values were calculated. The resulting models for DD and HC were compared. As we were especially interested in the factors affecting the “WANT” ratings, we tracked the path that led to and from “WANT” ratings in each group. Below, we will indicate the direction of the effect with arrows (“→”). For example, the statement “A→B” means “A causes/affects B.”

## 3 RESULTS

### 3.1 Demographic and clinical

Individuals with DD had significantly higher lifetime and current dimensional symptoms of depression than HC, but did not differ from them in age, BMI, gender composition, or hunger/appetite level measured by the VAS prior to the scan (see Figure S1 for distribution of the HRSD‐25 scores in individuals with DD and Figure S2 for distribution of the VAS scores in individuals with DD and HC). IQ was in the same normal range for both groups but was higher for the individuals with DD than HC (Table 1). Of 54 individuals with DD, 33 had between 1 and 5 comorbid diagnoses (Table 1). The majority of comorbid diagnoses were anxiety disorders (generalized anxiety disorder: *n* = 19, social anxiety disorder: *n* = 12, panic disorder: *n* = 8, phobia‐related disorders: *n* = 4, obsessive‐compulsive disorder: *n* = 2). Three individuals had attention deficit disorder, four had binge eating disorder, and one had unspecified eating disorder.

**TABLE 1:** Demographic and clinical characteristics

	HC	DD	statistics HC vs. DD
*N*	48	54	
Gender (number females)	36	43	*χ* ^2^(1) = 0.7, *p* = .4
DD diagnoses (MDD/PDD)	na	36/18	na
Age (years); mean (SE)	28.09 (0.91)	27.9 (0.87)	*t*(100) = 0.15, *p* = .88
BMI; mean (SE)	25.82 (0.65)	25.25 (0.59)	*t*(100) = 0.66, *p* = .51
BMI (range)	18.3–39.5	17.7–35.8	
Number of overweight/obese participants with BMI > 24.9 (total %)	15/8 (48%)	15/9 (44%)	χ^2^(2) = 0.16, *p* = .9
IQ (NART); mean (SE)	106.72 (0.82)	110.12 (0.99)	*t*(100) = −2.6, *p* = .01
VAS before scan; mean (SE)	24.18 (2.76)	22.11 (2.73)	*t*(67) = 0.53, *p* = .6
Current depression severity (HRSD‐25); mean (SE)	1.75 (0.3)	13.31 (1.01)	*t*(100) = −10.4, *p* < .001
Lifetime depression (MOODS‐SR); mean (SE)	2.1 (0.33)	18.56 (0.56)	*t*(100) = −24.54, *p* < .001
Illness onset (year of age); mean (SE)	na	15.15 (0.67)	na
Number of participants taking antidepressants	na	38	na
Number of participants taking mood stabilizers	na	3	na
Number of participants taking antipsychotics	na	3	na
Number of participants taking benzodiazepines	na	7	na
Number of participants taking stimulants	na	6	na
A mean number of psychotropic medications; mean (SE)	na	1.15 (0.13)	na
A mean total medication load; mean (SE)	na	1.5 (0.17)	na
Number of participants with comorbid diagnoses 1 comorbid disorder 2 comorbid disorders 3 comorbid disorders 4 comorbid disorders 5 comorbid disorders	na	33 13 10 7 2 1	na

*Note*. The table reports the mean and standard error of mean (SE) in parenthesis.

ns, not significant; na, not applicable.

When we controlled age, sex, and IQ on the relationship between BMI and lifetime depression severity, we found a significant positive partial correlation in DD (*r* = 0.25, *p* = .001), but not HC (*r* = −0.13, *p* = .14). More severe lifetime depression symptoms were observed in individuals with DD who had higher BMI, compared to their lower BMI counterparts.

### 3.2 Behavioral

#### 3.2.1 Pleasantness responses, RT, and “LIKE” and “WANT” ratings

We found a main effect of stimulus type (food vs. object), but not a group (i.e., the DD/HC status) or group‐by‐stimulus interaction effects, on percent of items identified as pleasant (*F*(1,100) = 88.4, *p* < .001) and RT during pleasantness rating (*F*(1,100) = 79.6, *p* < .001). More “pleasant” responses were given to food versus objects (*t* = 9.4, *p*‐FDR‐corrected < .001; pleasant food: 78.15(2.17)%; pleasant objects: 58.22(2.17)%). Participants responded faster to food versus object stimuli (*t* = −8.9, *p*‐FDR‐corrected < .001; food: 831.9 [17] ms, objects: 903.8 [17] ms). The mean “LIKE” or “WANT” ratings were not associated with group, BMI, or group‐by‐BMI interaction (estimated means for “LIKE” ratings: DD: 6.26 [0.17], HC: 6.49 [0.17]; estimated means for “WANT” ratings: DD: 4.32 [0.24], HC: 4.25 [0.24]).

##### Anticipation strategies

Across all participants, the verbal strategy was used by 69.3% participants during both food and nonfood items anticipation; the visual strategy was used by 58.4% participants during food anticipation and by 50.5% during nonfood items anticipation; the rule strategy was used by 59.4% for both food and nonfood object items; the sensory strategy was used by 34.7% for food and 28.7% for objects; and the memory strategy was used by 31.7% for food and 27.7% for objects. Less than 5% of participants tried to actively suppress thoughts about the cued categories of items. Less than 5% of subjects responded that they did not use anticipation strategies or did not try to mentally prepare during the task. Importantly, there was no significant association between the choice of a particular anticipation strategy with diagnostic status and BMI (all *p* > .05).

### 3.3 Neuroimaging

#### 3.3.1 Primary analyses

**FIGURE 2:**
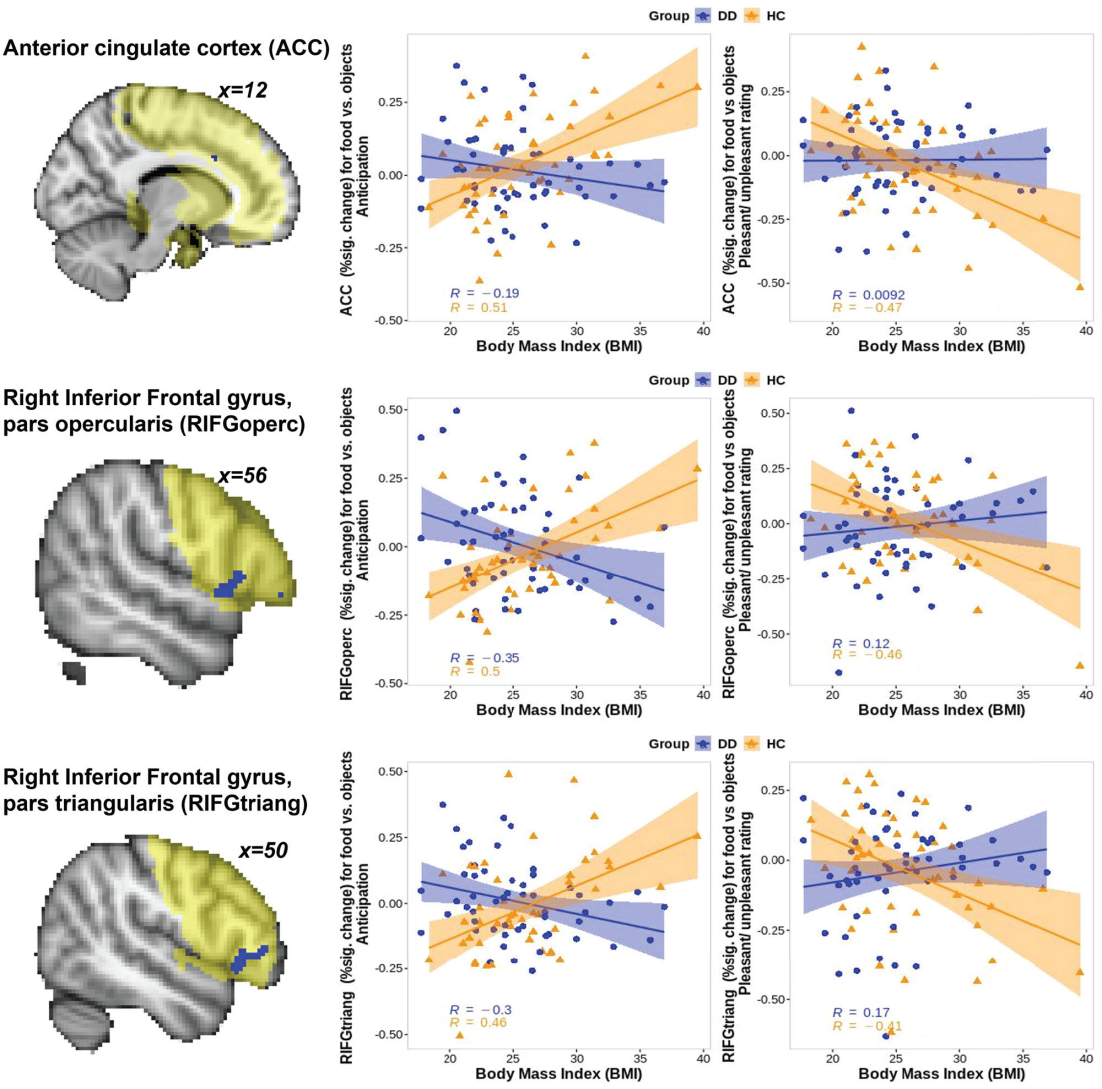
Group by BMI interaction effect on activation in the right inferior frontal gyrus pars triangularis and pars opercularis, and anterior cingulate cortex. The ROI mask is shown on the brain images in yellow, while the regions with a significant BMI‐by‐diagnosis interaction are shown in blue. The correlation coefficients and *p* values between BMI and brain activation for each group of participants are presented on each plot

A significant group‐by‐BMI interaction effect on brain activation during *anticipation* of food versus objects was found in the right inferior frontal gyrus pars opercularis (RIFGoperc; nvox = 56, *z*‐max = 4.86 [60, 16, 8]), the RIFG pars triangularis (RIFGtriang; nvox = 91, *z*‐max = 4.51 [48, 36, 0]), and a small cluster in the right anterior cingulate cortex (ACC; nvox = 5, *z*‐max = 5.36 [12, 16, 30]) (Figure 2).

While the main analyses did not reveal significant group‐by‐BMI interaction effect on brain activation during *pleasantness rating* for food versus objects, the follow‐up analyses conducted on the percent signal changes extracted from the RIFG pars triangularis, RIFG pars opercularis, and ACC showed a significant group‐by‐BMI interaction effect on brain activation during *pleasantness rating* for food versus objects in all these regions (RIFGoperc: *F*(1,95) = 8.8, *p* = .004; RIFGtriang: *F*(1,95) = 8.6, *p* = .004; ACC: *F*(1,95) = 8.5, *p* = .004). However, unlike the task *anticipation* condition in which HC showed positive, while DD showed negative, correlation between BMI and *anticipatory* brain activation in the ROIs above for food versus objects, *pleasantness* rating elicited a different pattern of activation. Specifically, a linear regression with age, sex, and IQ as covariates showed that there was a negative relationship between BMI and RIFGoperc, RIFGtriang, and ACC activation during *pleasantness* rating of food versus objects in HC (RIFGoperc: *t* = −3.5, *p* = .001; RIFGtriang: *t* = −3.5, *p* = .001; ACC: *t* = −4.3, *p* < .001), but no significant relationship between these variables in individuals with DD.

Considering the opposing pattern of the relationship between BMI and brain activation during anticipation and during pleasantness rating, we examined the correlations between *anticipation* and *pleasantness* rating for different categories of stimuli across all participants. We found a significant negative correlation between *anticipatory* and *pleasantness* rating activation for food versus objects in all regions (RIFGoperc: *r* = −0.64, *p* < .001; RIFGtriang: *r* = −0.63, *p* < .001; ACC: *r* = −0.84, *p* < .001), but no significant correlation between *anticipatory* and *pleasantness* rating activation for food and objects separately in all these regions (all *p* values > .1) with the exception of the positive correlation between these variables in the RIFGoperc for objects (*r* = 0.23, *p* = .022), which did not pass the Bonferroni correction threshold (0.05/9 = 0.0056).

##### Exploratory analyses

###### The effect of clinical variables on activation in the RIFG and ACC in DD

Of 54 individuals with DD, 4 reported having increase appetite, 19 reported decreased appetite, while 31 reported normal appetite. Of 48 HC, 3 reported having increased appetite, while 45 reported normal appetite. The food versus object differences in brain activation in RIFG regions and ACC during *anticipation* and *pleasantness rating* were not associated with medication load, age at illness onset, the number of depressive episodes, and appetite during the past week (all *p* values > .1) in DD. The HRSD‐25 scores measuring severity of current depression were not significantly associated with the “LIKE” or “WANT” ratings, or the differences in the food versus object in RT, pleasantness ratings, or activation in either ROI.

###### Graph modeling approach

**FIGURE 3:**
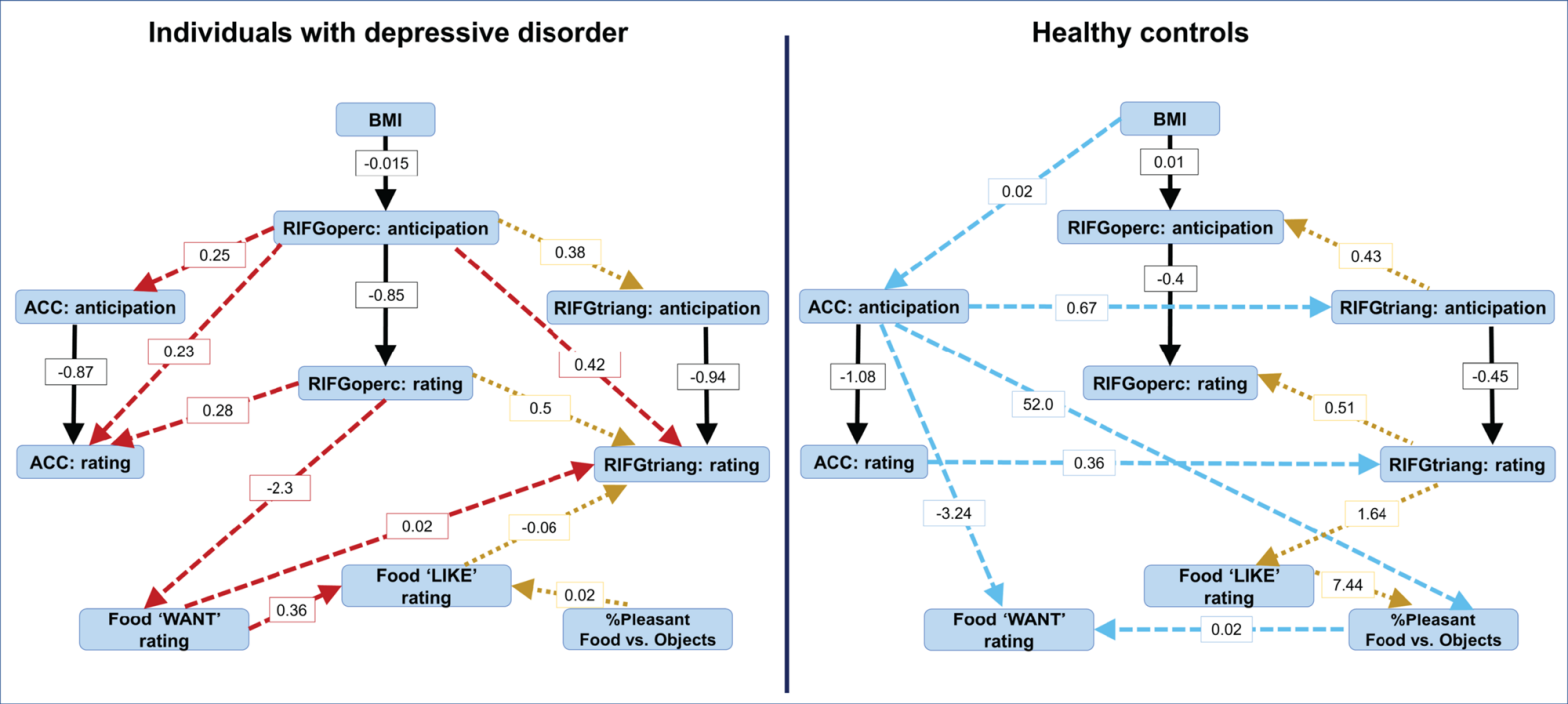
The relationship between BMI, brain, and behavior variables: graph models. The black solid arrows show the edges that connected the same nodes and had the same directions in DD and HC. The yellow dotted arrows show the edges connected the same nodes but having different directions for DD and HC. The red arrows show the edges unique for DD. The blue arrows show the edges unique for HC. RIFGoperc—right inferior gyrus pars opercularis, RIFGtri, right inferior gyrus pars triangularis, ACC—anterior cingulate cortex

All FGES outputs estimated separately in individuals with DD and HC (Figure 3) were DAGs (all edges were oriented). The same set of 10 nodes (variables) was used for both models and included BMI, activation in the ACC, RIFGtriang, and RIFGoperc regions during anticipation of food versus objects and during pleasantness rating of food versus objects, the differences in the percent of items judged as pleasant for food versus objects, and “LIKE” and “WANT” ratings. The model fit analysis suggested that both models were correctly specified (DD: degrees of freedom = 30, *χ* ^2^ = 22.6, *p* = .83, BIC = −97.1; HC: degrees of freedom = 31, *χ* ^2^ = 27.7, *p* = .64, BIC = −92.3). The model coefficients along with the *t*‐statistics and *p* values for each edge are reported in Table 2.

**TABLE 2:** Edge coefficients and statistics for the SEM PM

Variable 1 → Variable 2			DD		HC	
			Coeff	Statistics	Coeff	Statistics
BMI	→	RIFGoperc: anticipation	–0.015	*t* = −2.69, *p* < .01	0.01	*t* = 2.2, *p* < .05
ACC: anticipation	→	ACC: rating	–0.87	*t* = −9.43, *p* < .001	–1.08	*t* = −12.8, *p* < .001
RIFGoperc: anticipation	→	RIFGoperc: rating	–0.85	*t* = −8.2, *p* < .001	–0.4	*t* = −2.85, *p* < .01
RIFGtriang: anticipation	→	RIFGtriang: rating	–0.94	*t* = −8.5, *p* < .001	–0.45	*t* = −3.16, *p* < .01
RIFGoperc: anticipation	→	RIFGtriang: anticipation	0.38	*t* = 4.03, *p* < .001		
RIFGoperc: anticipation	←	RIFGtriang: anticipation			0.43	*t* = 3.63, *p* < .001
RIFGoperc: rating	→	RIFGtriang: rating	0.5	*t* = 4.66, *p* < .001		
RIFGoperc: rating	←	RIFGtriang: rating			0.51	*t* = 4.25, *p* < .001
Food “LIKE” rating	→	RIFGtriang: rating	–0.06	*t* = −4.05, *p* < .001		
Food “LIKE” rating	←	RIFGtriang: rating			1.64	*t* = 2.35, *p* < .05
%Pleasant food vs. objects	→	Food “LIKE” rating	0.02	*t* = 3.55, *p* < .001		
%Pleasant food vs. objects	←	Food “LIKE” rating			7.44	*t* = 2.55, *p* < .05
BMI	→	ACC: anticipation			0.02	*t* = 4.04, *p* < .001
RIFGoperc: anticipation	→	ACC: anticipation	0.25	*t* = 2.43, *p* < .05		
RIFGoperc: anticipation	→	ACC: rating	0.23	*t* = 2.13, *p* < .05		
RIFGoperc: anticipation	→	RIFGtriang: rating	0.42	*t* = 3.24, *p* < .01		
RIFGoperc: rating	→	ACC: rating	0.28	*t* = 3.02, *p* < .01		
RIFGoperc: rating	→	Food “WANT” rating	–2.3	*t* = −2.16, *p* < .05		
ACC: anticipation	→	%Pleasant food vs. objects			52.0	*t* = 2.79, *p* < .01
ACC: anticipation	→	Food “WANT” rating			–3.24	*t* = −2.8, *p* < .01
ACC: anticipation	→	RIFGtriang: anticipation			0.67	*t* = 4.75, *p* < .001
ACC: rating	→	RIFGtriang: rating			0.36	*t* = 2.65, 0.05
Food “WANT” rating	→	Food “LIKE” rating	0.36	*t* = 5.31, *p* < .001		
Food “WANT” rating	→	RIFGtriang: rating	0.02	*t* = 2.26, *p* < .05		
%Pleasant food vs. objects	→	Food “WANT” rating			0.02	*t* = 2.28, *p* < .05

## 4 DISCUSSION

In this study, we investigated how the interplay between depressive disorders (DD) diagnosis and BMI was associated with brain activation during *anticipation* and *pleasantness rating* of food versus object stimuli. Consistent with previous studies (Jantaratnotai et al., 2017; Luppino et al., 2010; Mannan et al., 2016; Mitchell et al., 2014), higher‐BMI individuals with DD had more severe lifetime depression. Activation in the RIFG pars triangularis, RIFG pars opercularis, and ACC was positively associated with BMI in HC, but negatively in individuals with DD, during *anticipation* of food versus objects. HC with higher BMI had greater food versus object differences (food > object) than HC with lower BMI. In contrast, among individuals with DD, those with higher BMIs had lower food versus object differences (food < object) than those with lower BMIs. The post hoc analyses of RIFG and ACC activation during *pleasantness rating* showed that higher‐BMI HC had lower (negative) activation in the RIFG and ACC regions for food versus objects (food < objects) than lower‐BMI HC. No such relationship was observed in individuals with DD. The results described above are not explained by participants’ level of hunger/satiation (Stockburger et al., 2008) because HC and DD did not differ in hunger/satiation level prior to the scan in our study. Consistent with recent findings that weight increase in mood disordered individuals was better explained by depression status rather than the use of antidepressants (Gibson‐Smith et al., 2016), the main outcomes in our study were unrelated to psychotropic medications load. They were also unrelated to age at depression onset, number of depressive episodes, and an increase or decrease in appetite (as per the HRSD‐25 assessment) during the past week. Our findings related to brain activation during *pleasantness rating* of food versus nonfood pictures were inconsistent with the results of the previous meta‐analysis showing greater activation in the RIFG and ACC regions for obese versus normal weight individuals for food versus nonfood stimuli (Brooks et al., 2013). This could be explained by the differences in the experimental paradigms as well as by the lack of formal assessment of mood disorders in participants included in that meta‐analysis.

The RIFG is involved in response inhibition and attention control (Aron et al., 2003; Garavan et al., 1999; Hampshire et al., 2010; Sebastian et al., 2016), which are necessary to regulate appetitive behavior. The ACC is engaged in multiple cognitive processes including anticipatory control (Aarts et al., 2008), anticipation of conflict monitoring (Sohn et al., 2007), prediction of future states based on chosen actions (Akam et al., 2021), and conflict monitoring (Barch et al., 2001; Botvinick et al., 2001). It shows greater activation in obese individuals with major depressive disorder compared to those without major depressive disorder during pleasantness rating of neutral words (Restivo et al., 2020). Our findings of distinct activation patterns in these regions for individuals with DD versus HC suggests that different brain mechanisms may underlie behaviors leading to overweight/obesity in these groups. The presence of DD diagnosis must be considered in weight loss intervention programs to improve outcomes.

Previous studies reported greater attentional bias to food stimuli in overweight/obese individuals (Yokum et al., 2011). Our study revealed high hedonic valence and emotional salience of food across all individuals. While preparing for and making judgments about food pictures is not necessarily more difficult than making such judgments about nonfood pictures, cognitive processing related to food may be more emotional. Given that processing of emotional information requires more attentional resources (Schupp et al., 2007; Vuilleumier, 2005), cognitive and neural mechanisms of mental preparation for processing of food stimuli may resemble the mechanisms of mental preparation for difficult tasks. In our study, HC with normal weight showed decreased activation in the RIFG and ACC regions during food versus objects *anticipation* but increased activation in these regions during *pleasantness rating*. This activation pattern resembles that observed during anticipation of and performance on the working memory task in which the prefrontal and paracingulate cortices decreased in activation during *anticipation* of a difficult versus easy working memory task, but increased in activation during *performance* on a difficult versus easy working memory task (Manelis & Reder, 2015; Manelis et al., 2020). Decreasing brain activation during anticipation of a more difficult task could be related to redistributing available cognitive resources by decreasing interference from the sources irrelevant to the current task during anticipation (Manelis & Reder, 2015). HC perhaps maintain normal weight by effectively preparing for food‐related stimuli processing thus controlling food consumption. In contrast, higher‐BMI HC might face greater cognitive challenge when anticipating an encounter with food: they engage the RIFG and ACC regions when no food stimuli are yet available but fail to engage these regions when encountering food stimuli, thus diminishing their ability to control appetitive behavior. Individuals with DD had an opposite pattern of relationship between BMI and activation during *anticipation* than HC and showed no significant relationship between BMI and activation during *pleasantness rating*. This suggests that individuals with DD need to establish attentional control and choose the course of actions prior to encounter with food by engaging the RIFG and ACC. Involving these regions during encounter with food is challenging for these individuals.

The exploratory graph theory analyses revealed similarities and differences between HC and DD graph models. We found that in both groups, BMI affected activation in the RIFGoperc during *anticipation* of food versus nonfood objects. However, higher BMI was associated with lower RIFGoperc activation during food versus object anticipation (negative association) in DD, but greater RIFGoperc activation during food versus object anticipation (positive association) in HC. In both groups, increases in anticipation‐related activation for food versus objects caused decreases in food versus object activation in the same region during pleasantness rating. However, the causal relationship between the RIFGoperc, RIFGtriang and ACC during anticipation and pleasantness rating was unique for each diagnostic group: in HC, the ACC influenced the RIFG regions (ACC→RIFGoperc→RIFGtriang) during both anticipation and pleasantness rating of food versus objects, while in DD, the changes in RIFGoperc caused the changes in RIFGtriang and ACC activation (RIFGoperc→RIFGtriang and RIFGoperc→ACC).

Using a graph theory approach in this study helps identify factors related to the “WANT” ratings as they might be a driving force for overeating. Making pleasantness judgments involves deep stimulus encoding (Richardson‐Klavehn, 2010; Schott et al., 2013), so the responses and brain activation during *pleasantness rating* of food items could be related to how much participants wanted to eat those food items. As we did not have a specific hypothesis about the causal relationship between “WANT” ratings and other variables and believed that participants could decide how much they wanted a particular food item either when they saw the item for the first time during the scan or during the post‐scan task, we allowed the FGES to discover connections between the “WANT” ratings and other variables. In HC, “WANT” ratings were regulated through the BMI → ACC: anticipation → “WANT” rating path as well as through the BMI → ACC: anticipation → percent of pleasant food versus object responses → “WANT” ratings path. The first path leads HC with higher BMI to report lower “WANT” ratings, while the second path leads them to report higher “WANT” ratings. Thus, a realization that food is more pleasant than nonedible items may result in increased drive to eat that food in HC. Remarkably, the “WANT” ratings did not affect any brain or other variables.

In DD, the “WANT” ratings were regulated through the BMI → RIFGoperc: anticipation → RIFGoperc: rating → “WANT” ratings path, so that higher‐BMI individuals with DD reported lower “WANT” ratings. Importantly, the “WANT” ratings affected activation in the RIFGtriang during food versus object pleasantness rating either directly or through the “LIKE” ratings. The direct path would lead to decreases in food versus object activation in the RIFGtriang if the “WANT” ratings were low. However, if participants did not want and like food items, then the RIFGtriang activation increased. The increases in brain activation in this region for food versus objects might indicate an attempt to exhibit attentional control during processing of food stimuli.

One may think that because anticipatory periods always preceded pleasantness ratings, the two regressors would always anticorrelate. We indeed found a strong negative correlation between anticipation and pleasantness ratings activations for food versus objects differences. However, there was no significant relationship between anticipatory and pleasantness rating activation when food and objects were analyzed separately. This suggests that observed effects for food‐object differences are unlikely due to some artifactual anticorrelation.

Comparing brain activation for the food stimuli rated as pleasant versus unpleasant would be potentially informative, but we were not able to examine this question: there were many more pleasant than unpleasant responses to food stimuli, with many participants rating all food items as pleasant. Future studies should address this limitation by presenting both appealing and nonappealing pictures of food as well as replicate the findings from graph model analyses. Another limitation concerns using self‐reported height and weight to calculate BMI. While overweight/obese people tend to underestimate the weight status of self and other people (Oldham & Robinson, 2018; Robinson, 2017), this bias is more pronounced in older than in younger adults (Kuczmarski et al., 2001). In addition, the participants in our study were not asked to estimate their weight status, and, instead, they were informed that height and weight are collected to determine MRI eligibility and safety. Considering that self‐reported height and weight were previously successfully used in the epidemiological studies (Carpenter et al., 2000; Simon et al., 2006), were shown to correlate with physical measurements (Cash et al., 1989), and were reliably used with participants younger than 60 years of age (Kuczmarski et al., 2001), we believe that self‐reported body size accurately reflected actual body size in our study. Finally, we were not able to recruit individuals with extreme obesity (BMI > 40) due to the scanner weight limit and the size of the radio frequency coil. These limitations can be overcome by employing functional near infrared spectroscopy (fNIRS) that has recently been successfully implemented in studies of obesity (Huang et al., 2019; Rösch et al., 2021) and depression (Manelis et al., 2019).

In summary, this study showed that the interplay between diagnostic status and BMI affects activation patterns in the RIFG and ACC regions during *anticipation* and *pleasantness rating* of food versus nonfood objects. Importantly, different neurobiological mechanisms underlying participants’ desire to eat food items presented in the study. Future clinical trials targeting weight loss in individuals with depressive disorders should investigate whether weight loss programs designed for these individuals should differ from those designed for HC by focusing on food anticipation in the former.

## CONFLICT OF INTEREST

A.M., Y.O.H., S.S., R.R., S.I., and M.D.L. declare no conflict of interest. H.A.S. receives royalties from Wolters Kluwer, royalties and an editorial stipend from APA Press, and honoraria from Novus Medical Education and Medscape.

## AUTHOR CONTRIBUTIONS

A.M. obtained funding, designed the study, acquired data, evaluated data quality, analyzed, and interpreted the data and drafted and critically evaluated the manuscript. Y.O.H. curated data organization and analyses, drafted, and critically evaluated the manuscript. S.I. curated data analyses and critically evaluated the manuscript. S.S. and R.R. acquired data, evaluated data quality, drafted, and critically evaluated the manuscript. H.A.S. curated participants’ recruitment, interpreted the data, and critically evaluated the manuscript. M.D.L. curated study development, interpreted the data, and critically evaluated the manuscript. All authors have read and approved the final version of the manuscript and agreed to be accountable for all aspects of this work.

### PEER REVIEW

The peer review history for this article is available at https://publons.com/publon/10.1002/brb3.2695

## Supporting information


**FIGURE S1**. Density distribution for the HRSD‐25 scores measuring severity of current depression in individuals with depressive disorders (DD)
**FIGURE S2**. Density distribution for the VAS scores measuring the level of hunger in individuals with depressive disorders (DD) and healthy controls (HC)

## Data Availability

Data available on request from the authors.
