# Supplementary material for: The interaction between depression diagnosis and BMI is related to altered activation pattern in the right inferior frontal gyrus and anterior cingulate cortex during food anticipation
Source: Brain Behav. 2022 Aug 12;12(9):e2695. doi: 10.1002/brb3.2695 (PMC9480896; doi:10.1002/brb3.2695)
Supplement: Supplementary file 1 — FIGURE S1. Density distribution for the HRSD‐25 scores measuring severity of current depression in individuals with depressive disorders (DD) FIGURE S2. Density distribution for the VAS scores measuring the level of hunger in individuals with depressive disorders (DD) and healthy controls (HC) [file BRB3-12-e2695-s001.docx]

# SUPPLEMENTAL MATERIALS

## Supplemental figures


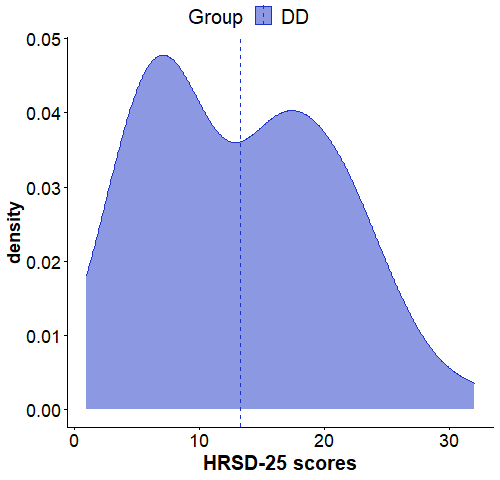


**Figure S1**. Density distribution for the HRSD-25 scores measuring severity of current depression in individuals with depressive disorders (DD).


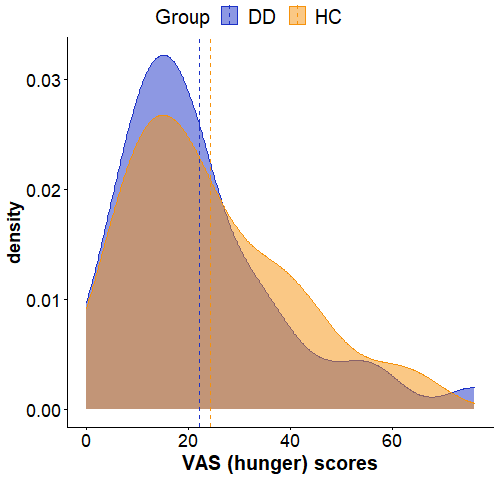


**Figure S2**. Density distribution for the VAS scores measuring the level of hunger in individuals with depressive disorders (DD) and healthy controls (HC).
